# Effect of body mass on future long-term care use

**DOI:** 10.1186/s12877-020-01688-4

**Published:** 2020-08-17

**Authors:** Olena Nizalova, Katerina Gousia, Julien Forder

**Affiliations:** 1 PSSRU, University of Kent, Canterbury, UK; 2 CHSS, University of Kent, Canterbury, UK; 3 School of Economics, University of Kent, Canterbury, UK

**Keywords:** Long-term care, Elderly people, Formal care, Social care, Informal care, BMI, Obesity

## Abstract

**Background:**

Obesity is a known predictor of disability and functional limitations, and, in turn, of health care use. In this study, we aim to explore whether obesity is also a significant risk factor for future long-term care use, overall and by type of care.

**Methods:**

We use multinomial logistic regression analysis on data from the English Longitudinal Study of Ageing (ELSA) for individuals aged 65 and older between 2002 and 2011. Selection issues are tackled using the rich set of control variables, exploiting the data’s longitudinal structure and accounting for loss to follow-up (including death). Control factors include health-related behaviours (physical activity, alcohol and tobacco consumption), functional limitations (related to ADLs, iADLs and mobility) and specific existing health conditions, notably diabetes, high blood pressure and cardio-vascular diseases.

**Results:**

We find that obese older people are 25% (*p* < 0.01) more likely to receive informal or privately-paid care in the future, but this does not hold for formal care. This is an additional direct effect after controlling for a wide range of health conditions and functional limitations. We document some evidence that this effect is due to the development of new functional limitations. Sensitivity analyses suggest that the results are robust to controlling for prediabetes, subjective health, depression, or unobserved heterogeneity.

**Conclusions:**

This study provides new evidence of a positive direct effect of obesity on the future use of long-term care services. Accordingly, it adds evidence of further economic benefits to any overall evaluation of policies to promote a healthy weight in the population, particularly in the older population.

## Background

In the UK, as in many other countries, the prevalence of obesity is rising to epidemic proportions. About 40% of Britons are projected to be obese by 2025, and Britain is projected to become a largely obese society by 2050 [1]. The impact of increased obesity on societies and governments has been significant [2, 3]; obesity is related to premature mortality [4] and is a risk factor for several chronic conditions, including type 2 diabetes [5, 6], cardiovascular diseases [7], some cancers [8, 9], osteoarthritis [10], hypertension, respiratory diseases and others [11]. Economic consequences are the substantial financial costs – via the need for additional medical services - associated with fatal and non-fatal obesity-related diseases [2, 3] (an estimated extra £5.5 billion for the UK National Health Service (NHS) by 2050 [1]) and other indirect costs, such as lost workdays, disability pensions, reductions in productivity and decreases in disability-free life years [12].

There is evidence that obesity is directly associated with functional limitations (e.g. mobility) and disability in old age; including from physical disabilities, increased cognitive impairment and reduced psychological well-being among older people [13–16]. This evidence, along with findings of an upward shift in the age at which body fat and body mass index stop increasing [17], suggests that there will be an increasing need for long-term care (LTC) in the future. However, the evidence on the impact of obesity on the use of LTC services and consequent costs is limited. A few US studies have sought to explore the direct relationship. Elkins et al. [18] found some evidence that obesity in mid-life is associated with a higher probability of nursing-home entry. Similarly, Zizza et al. [19], Resnik et al. [20] and Yang and Zhang [21] found that obesity in older people increases the risk of nursing-home admissions, use of personal care assistance and LTC costs.

**Fig. 1:**
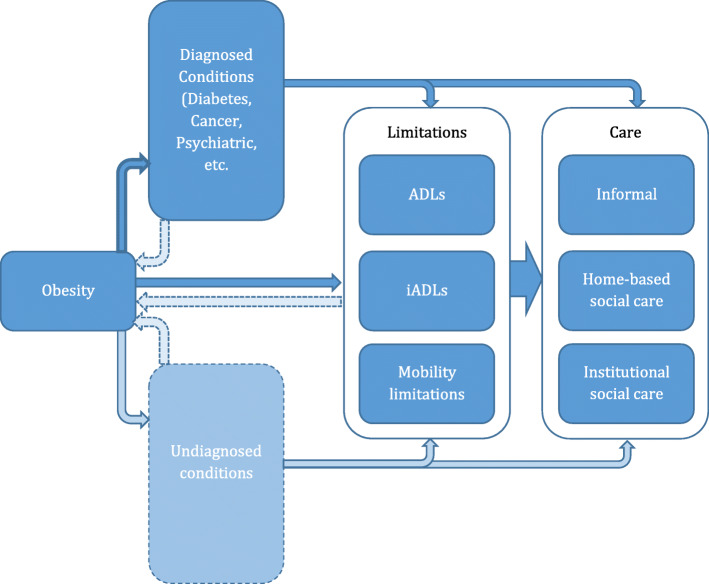
Pathways of obesity’s impact on future use of care

Our main aim is to estimate the effects of obesity on overall LTC use and, separately, on various types of LTC. We can consider the process by which obesity might have an impact. First, obesity is associated with diagnosed health conditions and other observed functional decline, which both increase the need for LTC. These two routes are depicted on the top part of Fig. 1 with solid dark arrows. We hypothesise that, in addition to this, we can observe a direct effect of obesity on future LTC use stemming from (as yet) undiagnosed conditions, further unmeasured functional limitations or non-health factors – as shown in the bottom section of Fig. 1. In particular, that even after controlling for observed health conditions and impairment, we still may see an independent effect from obesity on future care use that may or may not be related to measured health status. We also note in Fig. 1 the potential for certain diseases and functional limitations to be *causes* of obesity, recognising issues with establishing causal effects from obesity on the need for care with light dashed arrows.

An estimate of future LTC use that is attributable to obesity beyond currently known indicators of impairment will prove useful for decision making in both public health and social care. First, this allows us to account for a wider range of benefits from policies to tackle obesity in decision-making and, thus, reach more socially optimal levels of investment in corresponding interventions. Second, if obesity serves as a signal for impairment and future care needs that is not yet diagnosed or assessed, then accounting for increases of obesity prevalence in population would further help to improve planning and budgeting processes, and allow for better targeting of care-system resources in the future. In our analysis we focus on people aged 65 and up in England, as this population group is most at risk of requiring LTC and is more likely to be using (expensive) institutional care. We use a nationally representative dataset to estimate the effect of current obesity status on the future use of various types of care.

## Methods

### Social care context

Long-term care support for adults with chronic health conditions and disabilities usually comprises nursing care, personal care and assistance with domestic tasks [22]. Care can be provided either formally through professional services paid by individuals or local authorities or informally by family members, friends and neighbours [23]. Formal LTC, or more generally, social care as it is known in England, is provided by voluntary organisations, local councils, health authorities and private agencies and includes a range of services such as institutional care in care (nursing) homes, home care, day care, meals on wheels and others. England has a means-tested formal social care system whereby a publicly-funded ‘safety-net’ is in place for those with eligible financial circumstances and with greater need [24]. For our purposes we define ‘informal’ care as being provided by unpaid carers (e.g. often family members). Approximately 85% of all older people with a functional disability living in private households in England receive some informal care [22]. The number of informal care providers has increased over the years (by 11% between 2001 and 2011) and informal care itself has become more intensive, and, according to some estimates, reaching the total annual value of £55 billion [23].

### Data

Data is taken from the English Longitudinal Study of Ageing (ELSA), which is a longitudinal, biennial survey of individuals aged 50 and over. It was originally sampled from the pool of respondents to the Health Survey of England (1998, 1999, 2001) and collects data on individual and family circumstances and quality of life. We pooled data from waves 1 to 5.

In our analysis, the dependent variable is a categorical indicator measuring either any care or types of care, and non-response outcomes, including death. Care use was indicated where the survey respondent answered that they had help with activities affected by functional limitations from the associated question or were living in a care institution. Answers to further questions were used to determine the type of care used - see details in Table A1, Additional file 1: Appendix A. To avoid inconsistencies with the correspondence of categories across waves and to ensure a reasonable share of cases per category, we aggregated to broader care categories.

In line with the existing literature, our main indicator for *obesity* is derived from the body mass index (BMI) calculated from height and weight clinically measured by the nurses to the nearest millimetre and the nearest 0.1 kg respectively. Compromised measurements have been set to missing values [25]. We classify respondents into four groups according to the World Health Organisation’s (WHO) definition: underweight (BMI less than 18.5), normal weight (BMI 18.5 to 24.99), overweight (BMI 25 to 29.99) and obese (BMI of 30+). BMI was calculated directly for waves 2 and 4, and imputed for wave 1 (using wave 0 data). This was used as a risk factor for outcomes for waves 2, 3 and 5 respectively. Excluding the data from waves 0/1 does not change the main results, however it does prevent us from analysing heterogeneous effects due to the small sample size.

We use four sets of control factors [26–29]:

(i) Demographic, situational and economic (respondents’ age, number of children, real-per-capita total household income and wealth, and indicators as to whether a respondent is female, has no educational qualifications, or is non-white, married, living alone, or owns his/her home, and time dummies);
(ii) functional limitations (number of limitations with activities of daily living (ADLs), e.g., dressing, washing, transfer; with instrumental activities of daily living (iADLs), e.g., shopping and meal preparation; and with mobility, e.g., walking 100 yards);
(iii) variables describing health-related behaviours (indicators for alcohol drinking, cigarette smoking and physical activity). Physical activity was defined as indicated if an individual had responded in the associated question that they engaged in either: (i) vigorous physical activity at least one to three times per month or more often; (ii) moderate physical activity at least once a week or more often; or (iii) light physical activity more than once per week;
(iv) variables describing specific health conditions, such as high blood pressure, diabetes, cancer, lung disease, heart-related problems, stroke, psychiatric disorders and arthritis.

### Analysis

The analysis was performed using Stata 15. We started with the descriptive analysis and using locally weighted regression (LOWESS) to gain insights into the functional relationship between care use and BMI.

The main models were estimated to account simultaneously for a range of outcomes, including the various types of long-term care support, no support, non-response and death:

1 $$ \mathit{\ln}\left(\frac{p_{it j}}{p_{it1}}\right)={\beta}_{0j}+{W}_{it-1}{\beta}_{1j}+{X}_{it-1}{\beta}_{xj}+{\varepsilon}_{it} $$

For person *i* at time (wave of the ELSA survey) *t*, *p*_*itj*_ = *prob*(*y*_*itj*_|*X*_*it* − 1_, *W*_*it* − 1_) is the probability that the individual experiences outcome *j*. In the baseline specification, *j* includes (i) any type of care, (ii) non-respondent and (iii) dead. In the extended specification, *j* includes (i) informal care, (ii) informal and privately-paid care and (iii) formal care (care home and LA social care), (iv) non-respondent and (v) dead. The base category in both models is no use of care. We exploited the longitudinal nature of the data (ELSA has waves 2 years apart) in an attempt to mitigate any contemporaneous bias from unobserved confounding factors that have a short-term effect (for example, a person’s current level of self-confidence, which is unobserved, might affect both the need for LTC and obesity in the current period, but is less likely to be correlated with past obesity). As such in this analysis, the person’s care use in the current period *t* is specified to depend on their obesity status, *W*_*it* − 1_, and the other controls, *X*_*it* − 1_, as measured in the previous period (wave). Additional file 1: Appendix B provides details on setting up the econometric model and strategy of dealing with bias in more detail.

These models were estimated using multinomial logit. Standard errors were clustered at the individual person level. As a robustness check, to account for the unobserved time-invariant individual effects, we also estimated an alternative specification with a quadratic function in BMI using the unobserved effect logit model (xtmelogit in Stata).

## Results

### Descriptive statistics

Table 1 presents summary statistics for the main sample used in the analysis, as a whole and by type of care. Overall, in the whole sample we used, 30% of respondents receive some type of care in the future, of which 1% are in care homes, 27% receive informal care, 2% receive formal care and 4% paid for care privately. BMI measures for people not receiving care of any type are lower than in the overall sample, as is the obesity prevalence. The obesity prevalence is highest for those receiving informal care (36% compared to 27% in the whole sample; *p* < 0.01). However, the prevalence of being overweight is highest for those not receiving any type of care. Summary statistics for control variables are available in Additional file 1: Appendix C, Table C1.

**Table 1:** Summary statistics for key variables

	Whole sample	No care	Informal care (only) IC	Informal and privately-paid care IC + PC	Formal (care home/ LA care) FC (CH + LA)	Non-response	Died
	(1)	(2)	(3)	(4)	(5)	(6)	(7)
No. of observations	12,323	7041	2504	347	187	1561	683
Any mode of care	0.30	0.00	1.00	1.00	1.00	n/a	n/a
Informal care	0.27	0.00	1.00	0.31+	0.56^a^	n/a	n/a
Privately-paid care	0.04	0.00	0.00	1.00	0.09^a^	n/a	n/a
Formal (LA care)	0.02	0.00	0.00	0.00	0.95^a^	n/a	n/a
Formal (care home)	0.01	0.00	0.00	0.00	0.10^a^	n/a	n/a
Underweight (t-1)	0.01	0.01^a^	0.01	0.02	0.02	0.01	0.03^a^
Overweight (t-1)	0.44	0.47^a^	0.39^a^	0.38^b^	0.36^b^	0.41^b^	0.40^b^
Obese (t-1)	0.27	0.24^a^	0.36^a^	0.33^b^	0.32	0.27	0.23^a^
BMI (t-1)	27.73	27.41^a^	28.81^a^	28.11	28.52^b^	27.61	26.87^a^
	[4.77]	[4.28]	[5.40]	[5.51]	[6.38]	[4.88]	[5.29]

^a^indicates that the average for a specific category is statistically different from the average for the whole sample at a 1% level of significance, ^b^ - at the 5% level and + − at the 10% level

**Fig. 2:**
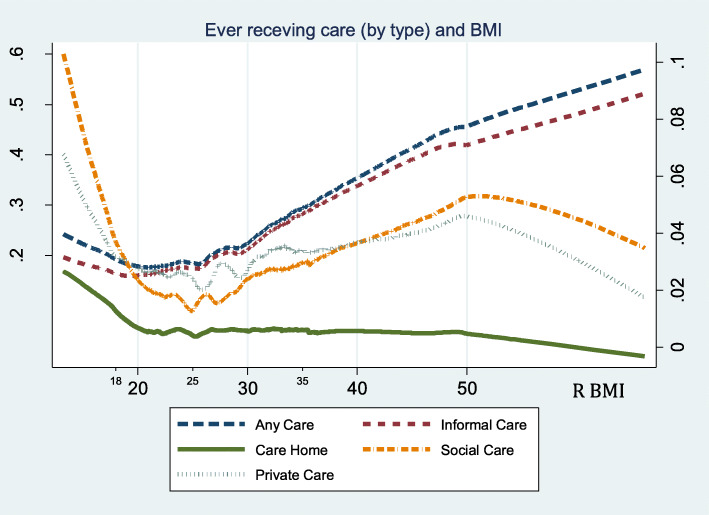
Non-parametric relationship between BMI and future use of care. Note: Any Care and Informal Care are on the scale of the left-hand y-axis, Care Home, Social Care and Private Care are on the scale of the right-hand y-axis

Figure 2 reports the (LOWESS) analysis of the relationship between care use and BMI. It shows that individuals with higher BMIs are far more likely to use care in the future, except for being in the care home.

### Any care specification

Table 2 reports the main results for the any-care estimation specified in eq. (1). The coefficients in the table are relative risk ratios, with (clustered) standard errors in parenthesis (full estimation results are in Additional file 1: Appendix C, Tables C2-C3). We estimate various specifications to explore the impact of the inclusion of additional controls on the magnitude of obesity’s effects on future care use. Panel A features the results for the full sample of people aged 65 and up, with respondents who do not use any care being the base category. Panel B restricts the sample to the individuals who receive no care of any type at the start of the period. As reported in column (1) in Panel A (the whole sample), obese people, compared with people of normal weight, are 75% more likely (*p* < 0.01) to use some care in the future (controlling for death and non-response). If we add controls for health behaviours such as physical activity, smoking and drinking (column [2]), the effect’s magnitude decreases somewhat, but still remains significant at 65% (*p* < 0.01).  Column 3 reports the results as we add demographic and socioeconomic controls, as well as ADL, iADL and mobility limitation-counts in the third specification, the effect of obesity decreases further, but still remains statistically: obese individuals are 28% more likely (*p* < 0.01) to use care in the future compared to individuals at a normal weight.

**Table 2:** Simple vs. Extended model results

	Basic Model (Any Care)				Extended Model (Full controls)		
	(1)	(2)	(3)	(4)	IC (5)	IC + PC (6)	FC (CH + LA)(7)
Panel A. All respondents ages 65 and up (*N* = 12,323)							
Underweight	1.78* (0.42)	1.57+ (0.37)	1.32 (0.35)	1.36 (0.36)	1.28 (0.36)	1.53 (0.83)	1.73 (1.02)
Overweight	0.93 (0.06)	0.96 (0.06)	0.98 (0.06)	0.96 (0.06)	0.96 (0.07)	0.99 (0.15)	1.02 (0.20)
Obese	1.75** (0.12)	1.65** (0.11)	1.28** (0.09)	1.25** (0.09)	1.26** (0.10)	1.27 (0.21)	1.16 (0.26)
Panel B. Respondents age 65 and up with no care initially (*N* = 8770)							
Underweight	1.77+ (0.58)	1.66 (0.54)	1.41 (0.47)	1.44 (0.49)	1.42 (0.53)	1.16 (1.09)	1.90 (2.13)
Overweight	0.99 (0.08)	0.99 (0.08)	0.97 (0.08)	0.93 (0.08)	0.92 (0.09)	1.20 (0.27)	0.68 (0.25)
Obese	1.71** (0.15)	1.65** (0.14)	1.34** (0.13)	1.27* (0.12)	1.30* (0.13)	1.32 (0.34)	0.80 (0.35)
Controls:							
Health behaviours	No	Yes	Yes	Yes	Yes		
Socio-demographic _ functional limitations	No	No	Yes	Yes	Yes		
Diagnosed health conditions	No	No	No	Yes	Yes		

** indicates significance at 1% level, * at 5% level and + at 10% level

In addition to the specified controls, all regressions include time dummies, and standard errors clustered at individual levels

**Table 3:** Relative risk ratios from multinomial logit––sensitivity check with basic model

	Basic Model (Any Care)					
	(1)	(2)	(3)	(4)	(5)	(6)
Underweight	1.36 (0.36)	1.40 (0.39)	1.73 (1.10)	1.28 (0.34)	1.25 (0.33)	2.03* (0.63)
Overweight	0.96 (0.06)	0.98 (0.07)	1.09 (0.18)	0.97 (0.06)	0.97 (0.07)	0.95 (0.08)
Obese	1.25** (0.09)	1.34** (0.11)	1.70** (0.30)	1.24** (0.09)	1.24** (0.09)	1.15 (0.11)
**Added/excluded controls**						
Full controls for health and health behaviours	Yes	Yes	Yes	Yes	Yes	Yes
Abdominally obese (AO)		1.15* (0.07)				
Pre-diabetes			1.47 (0.37)			
Self-rated health good or better				0.60** (0.04)	0.59** (0.04)	
CESD score					1.00 (0.02)	
Concurrent characteristics						
N ADLs						1.08 (0.07)
N iADLs						4.09** (0.31)
N of mobility limitations						1.41** (0.03)
N obs	12,323	10,794	2874	12,319	12,322	10,075

Notes: In addition to the specified controls, all regressions include time dummies, and standard errors clustered at individual levels. ** indicates significance at 1% level, * at 5% level and + at 10% level.

Column (4) of Table 2 presents the specification that includes a full set of health risk factors, such as high blood pressure, diabetes, cancer, lung and heart problems, stroke, psychiatric problems and arthritis. As can be seen, the effect has decreased further, while still remaining statistically significant: an obese person is around 25% more likely (*p* < 0.01) than a person at a normal weight to be using some type of care in the future.

Comparing the results (in Table 2) for the full sample (Panel A) and those for the restricted sample of individuals starting with no care (Panel B), we found that the estimates for the variable of interest become slightly larger in the specification with full set of controls, but are still statistically significant at the 5% level. 

### Extended specifications

Rather than outcomes categorised as any care (or not), plus non-response and death, the analysis was also conducted using an extended set of outcomes – for various types of care. Columns (5)–(7) in Table 2 (panel A) show results in which categories are defined according to types of care: (i) only informal care (IC); (ii) informal and privately-paid care (IC + PC); and (iii) formal care (both care homes and social care provided by Local Authorities) (FC). Respondents who receive the latter type of care are grouped in this category regardless of their use of informal or privately-paid care.

The overall impact of obesity on any care-use appears primarily due to the effect on informal care, while the effect on privately-paid care or formal care is smaller (16% (*p* > 0.05) compared with 26% (*p* < 0.01)) and not statistically significant. However, the latter may be due to the relatively low number of cases in this category (see Table 1 for descriptive statistics).

Potentially, respondents’ current care status may be driving the effect on the future care use. To test this, we ran all the specifications on the sample restricted to those who did not use any care initially (see panel B in Table 2). We found almost no qualitative difference in the results between the two samples. If anything, the effect was larger in magnitude for the sample with no initial care use.

We also assessed whether the effect sizes regarding obesity differ by gender. When estimating models with interaction terms on these variables, we found no statistically significant difference between genders with regards to obesity effects (results are available upon request).

### Sensitivity analysis

To assess robustness to different model specifications, we estimated a range of alternatives (see Table 3). For easy reference, column (1) repeats the results from the main specification with full set of controls (column (4) in Table 2).

First, we investigated the use of the BMI-based obesity measures vs. an abdominal obesity (AO) measure. If AO better captures the risk for future care use, we expect to see a further decline in the magnitude of the coefficient on general obesity indicator. The AO indicator is calculated based on the waist-hip ratio (WHR),^1^ which was available for a sub-sample of the data.^2^ We found that controlling for AO did not reduce the magnitude and significance of the main coefficient of interest – on the contrary, it became larger in magnitude with obese individuals being 34% more likely (*p* = 0.01) to use some type of care in the future (column (2) in Table 3). Moreover, the results suggest that regardless of the BMI-based obesity status, having abdominal obesity means being a further 15% more likely (*p* < 0.05) to use care in the future. Moreover, this effect is preserved when we drop the BMI-based obesity measures from the specification (results are available upon request), suggesting an independent AO effect on future use of care, which merits further investigation.

Second, we considered pre-diabetes as an explanation for the obesity effect we found. ELSA contains data on blood sugar levels for around a quarter of the sample,^3^ from which we calculated a ‘pre-diabetes’ indicator using fasting blood glucose levels.^4^ As column (3) in Table 3 reports, we found that pre-diabetes increases the probability of using care in the future, but it is not statistically significant. At the same time, while controlling for it, obese individuals are now 70% more likely (*p* < 0.1) to use some type of care in the future. However, these results should be treated with caution given a significant drop in the number of observations in the blood sample.

Third, we explored subjective health and depression as further explanations for the obesity effect, where in the main analysis we focussed mostly on the functional limitations and health conditions diagnosed by a doctor as major determinants of care. In this way, we examined the effect of having good or better self-rated health and also of depression – using the Centre for Epidemiologic Studies Depression scale – as proxies for other yet-to-be-diagnosed health conditions. As reported in columns (4)–(5) in Table 3, when various combinations of these control factors were specified in the main model, we found no difference from the main result regarding the effects of obesity, while the effect of having good or better self-rated health reduces use of care in the future and depression has no effect at all.

**Fig. 3:**
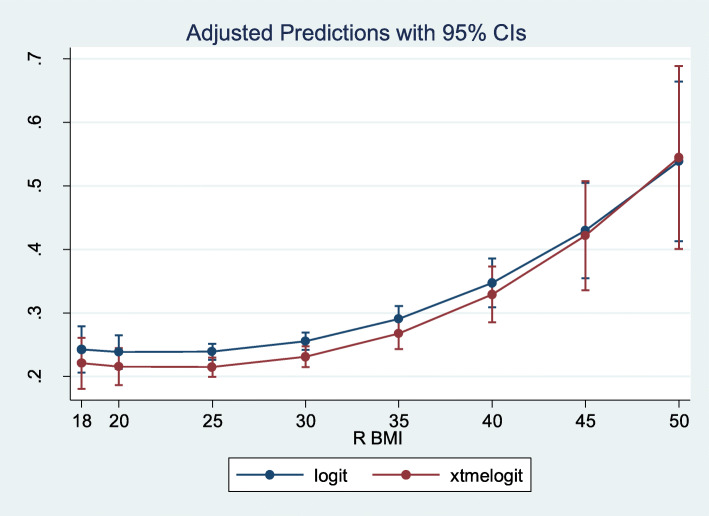
Predicted probability of future care use from the model with and without unobserved effects

Finally, column (6) reports the estimates from a regression in which the concurrent counts of ADLs, iADLs and functional limitations were used, i.e., not lagged with respect to the outcome measure. Their inclusion reduced the significance and the magnitude of the obesity’s effect. We might expect the current need for care to be highly correlated with current impairment rates (essentially by definition). Indeed, (lagged) obesity does not appear to affect care need beyond its effect on impairment rates. We also explored the consequences of using different estimators as noted above (unobserved effect logit model, using a quadratic function in BMI). Figure 3 reports the results – we find no statistically significant difference in the predictions from the two models.

## Discussion and conclusions

The rising trend in the prevalence of obesity presents a challenge for future health care and social care needs. Although the impact on health care has received more attention, the implications from obesity in relation to LTC utilisation and costs are not yet well understood.

Using data from the English Longitudinal Study of Ageing and a cohort study design, we found a significant association between obesity indicators and care use. Control factors included health-related behaviours, various health conditions, ADLs, iADLs and mobility limitations, with the analysis also accounting for attrition due to non-response and death. This result was in keeping with the few studies in this area of which we are aware [18–21].

In line with existing literature [5–11], we expected obesity to be a risk factor for several long-term conditions (e.g., diabetes, arthritis, heart failure, etc.), as well as a cause of impaired functioning in everyday life through ADLs, iADLs and mobility limitations [14–16]. Loss of functioning from either cause would increase the need for (and the benefits from) LTC. As such, observed indicators of long-term conditions (e.g., reported/diagnosed chronic diseases) and impairment (e.g., reported failure to achieve ADLs) should be associated with increased use of services, all other things being equal.

We also hypothesised that obesity could be an independent, direct risk factor for future care use, even where these observed indicators were used in the estimation, for three reasons: first, because obesity is a proxy for undiagnosed/unobserved health conditions; second, because disability and ‘need’ are in part socially constructed so that being obese implies a need for care (potentially beyond the actual health need); and third, because assessment of need is imperfect and could put too much weight on overt indicators like obesity.

Further analysis explored the effect on different components of care (in addition to the analysis of any-care effects). We found that much of the any-care effect was accounted for by the effects on informal care use specifically, although we need to be aware of modelling limitations when estimating the effect on particular types of care (see below).

In keeping with our expectations, where account was made of the effects of other conditions and impairments (as measured by ADLs, iADLs and mobility limitation indicators) on the use of LTC, the remaining direct obesity effect on LTC-use was smaller but still significant. Indeed, the remaining obesity effect size was less than half of the effect without controls, underlining the important (and strongly hypothesised) mediating effects of impairment. Nonetheless, a clear direct effect remained in our analysis.

As with observational analyses of this kind, we cannot rule out that these results might be partly explained by some other unobserved factor. However, we have included controls for the most theoretically likely factors and have taken some steps to minimise the potential for omitted variables bias – see limitations below.

Increasing obesity rates, among other things, imply greater care costs. An estimation of these costs can provide a sense of the obesity epidemic’s economic implications. As outlined, the primary effect we found of obesity was on the need for additional informal care. To illustrate the economic effects, we used our headline result (a 125% relative risk ratio of needing informal care if obese) and available estimates of the unit of costs of informal care [23], population structure [30], and the progression of obesity through time in the population [1]. We calculated on this basis that the ‘excess cost’ of obesity on LTC-use amounts to £3.9 billion for England for 2011 and £4.3 billion in 2013 (corresponding to the waves of ELSA used in this study). For comparison, Scarborough et al. [31] estimate the direct cost of both overweight and obesity to the NHS at £5.1 billion per year.

There are a number of limitations to this study. First, the well-rehearsed limitation of the multinomial logit model is the assumption on the independence of irrelevant alternatives (IIA). Several tests exist (most of which are incorporated into Stata routines), albeit not without limitations [32, 33]. The tests for the basic model with ‘any care’ category mostly supported the IIA assumption. The results for the extended model with several care categories turned out to be more problematic, as the tests in most specifications rejected the independence of other alternatives. Alternative estimators for the extended model specification that do not rely on IIA assumptions are computationally intensive and were not feasible, given the relatively low number of cases in the privately-paid and formal care categories.

Second, the nurse visits to take clinical measurements only happened every other wave (i.e. every 4 years), a long enough period for significant changes in the BMI to occur, which would not be picked up in our study. This has also affected our sample size as we had to use only three waves of data, and, therefore, precluded some of the sensitivity analyses we could undertake.

Third, as all the variables, except for the measures of height and weight, are self-reported, it is important to consider the implications of the potential (measurement) error for the findings. With regards to the measurement error in the dependent variable, it does not represent a threat to the consistency of the estimates as long as the measurement error is not systematically related to one or more of the explanatory variables ([34]: p. 293). Things are more complicated with measurement error in the independent variables. In the case where measurement error is correlated with the unobserved factors, we are facing a possibility of attenuation bias ([34]: p. 295). This means that we are more likely to find coefficients being statistically not different from zero. It is also not clear what would be the implications of this for the coefficients on the variables which are not measured with error. However, the fact that our main variables of interest are constructed from the variables that are clinically measured by nurses, gives us some reassurance that any inconsistency from the measurement errors for the coefficients of interest would be minimal.

Finally, unobserved control factors may exist that are associated with, but not caused by, obesity. Certain (pre-existing) conditions might cause obesity, as well as the disabilities that give rise to long-term care needs. Possible examples might include vitamin D deficiency or personality traits such as self-confidence/independence and willingness-to-cope, which we could not control for. We used lagged obesity measures to mitigate (short-term) endogeneity issues. Moreover, our test for the differences between the results with and without accounting for the unobserved effects reveals that the coefficients of interest are not affected.

To conclude, this study provides new evidence of the impact of obesity on future long-term care costs and provides a rationale for both taking into account obesity trends when planning for future LTC spending and for adding these LTC effects to the economic benefits, further to those relating to health care and other services, when considering policies to promote a healthier weight.

## Supplementary information

**Additional file 1:** Appendices.

## Data Availability

The datasets used and/or analysed during the current study is available from the UK Data Archive upon registration. All the information about the survey and how to access it is available at https://www.elsa-project.ac.uk/. The data were made available through the UK Data Archive. ELSA was developed by a team of researchers based at NatCen Social Research, University College London and the Institute for Fiscal Studies. The data were collected by NatCen Social Research. Funding was provided by the National Institute of Ageing in the United States and a consortium of UK government departments co-ordinated by the Office for National Statistics. The developers and funders of ELSA and the Archive do not bear any responsibility for the analyses or interpretations presented here.

## Footnotes

1 WHO classifies abdominal obesity as having a waist-hip ratio above 0.90 m for males and above 0.85 m or females (https://en.wikipedia.org/wiki/Waist%E2%80%93hip_ratio#WHO_protocol).

2 We also ran the main regression using the same sub-sample, which produced qualitatively similar estimates for the coefficients on the variables of interest.

3 We also ran the main regression using the same sub-sample, which produced qualitatively similar estimates for the coefficients on the variables of interest, but significantly larger in magnitude – closer to the one reported in the regression with pre-diabetes.

4 A fasting blood sugar level from 5.6 to 7.0 mmol/L as per Mayo clinic recommendations https://www.mayoclinic.org/diseases-conditions/prediabetes/diagnosis-treatment/drc-20355284.
