# Supplementary material for: Effect of body mass on future long-term care use
Source: BMC Geriatr. 2020 Aug 17;20:293. doi: 10.1186/s12877-020-01688-4 (PMC7433070; doi:10.1186/s12877-020-01688-4)
Supplement: Supplementary file 1 — Additional file 1: Appendices. [file 12877_2020_1688_MOESM1_ESM.docx]

# Body mass and future long-term care use

# Appendix A:

Table A1: Wave Correspondence on Care Incidence.

| Variable | Questions in waves 1-2 | Questions in waves 3-5 |
| --- | --- | --- |
|  |  |  |
|  | (1) | (2) |
| Any care received = 1 if answering ‘yes’ to at least one of the Qs | 1. Individual outcome code (if in institution)  2. ‘Thinking about the activities that you have problems with, does anyone ever help you with these activities (including your partner or other people in your household)?’ | 1. Individual outcome code (if in institution)  2. ’Functioning: whether ever has help with mobility, ADL, IADL’ |
|  | ‘Who helps you with these activities?’ | ‘Whether receives help moving around house (wash/dress, preparing meals/eating, etc.) from…’ asked individually |
| Informal care received | - husband/wife - mother/father - son - son-in-law - daughter - daughter-in-law - sister - brother - grandson - granddaughter - other relative - friend/neighbour - other person - unpaid volunteer | - spouse or partner - parent - son - son-in-law - daughter - daughter-in-law - sister - brother - grandson - granddaughter - other relative - friend/neighbour - other person - volunteer organisation |
| Formal care received | - social or health service worker | - social services/LA arranged care - nurse - other health or social services |
| Privately paid care | - privately paid employee | - privately arranged care |
| Nursing home care received | Derived from respondent’s individual outcome code | Derived from respondent’s individual outcome code |

# Appendix B: Econometric Methodology

We start with a (linear) model:

|  | $y_{itj}=\alpha_{0}+\alpha_{1}W_{it}+\alpha_{2}x_{it}\left( W_{it} \right)+e_{it}$ | (1) |
| --- | --- | --- |

where $y_{itj}$ is the outcome which, in this case, is the utilisation of long-term care (of type $j$, e.g., informal care or formal home care) by person $i$ at time $t$. In theory, utilisation is a function of a set of risk factors, including whether the person is obese (written as elements in the vector $W_{it}$), other needs-related risk factors ($x_{it}),$ such as the prevalence of chronic conditions, as well as a set of ‘other’ factors ($e_{it}$). The $\alpha$’s are the coefficients that measure the size of risk factors’ effects on care use. Moreover, we assume that the other needs-related risk factors for long-term (LTC) use are also partly dependent on the person’s obesity, i.e., factors $x_{it}$ are functions of $W_{it}$.

In practice, not all relevant risk factors are available in the data, as some are unobservable. Suppose we re-write (1) as:

|  | $y_{itj}=\alpha_{0}+\alpha_{1}W_{it}+\alpha_{21}X_{it}\left( W_{it} \right)+\alpha_{22}Z_{it}\left( W_{it} \right)+e_{it}$ | (2) |
| --- | --- | --- |

in which $\alpha_{2}x_{it}\left( W_{it} \right)=\alpha_{21}X_{it}\left( W_{it} \right)+\alpha_{22}Z_{it}\left( W_{it} \right),$ with $X_{it}$ being observable risk factors, such as reported long-term conditions, and $Z_{it}$ being unobservable risk factors (e.g., behavioural responses/preferences).

This specification presents several econometric challenges. First, we need to be clear about the different ways that obesity could affect LTC use, both as a direct effect captured by $\alpha_{1}$ in (2) and indirectly, in which obesity status affects other factors that are included in the estimation, e.g., receiving a diabetes diagnosis or limitations with activities of daily living (ADLs) that stem from being obese (which are factors in $X_{it}$). The latter is captured partly in the coefficient vector $\alpha_{21}$. Second, any unobserved risk factor that is also correlated with the person being obese or not will bias the estimated coefficients in a standard (OLS) estimation of $y_{itj}$ on $W_{it}$ and $X_{it}$. We cannot be certain that obesity’s estimated effect is the actual causal effect or whether it also is capturing some effect from an unobserved factor that happens to be correlated with obesity’s prevalence (e.g., the person’s inherent self-confidence).

This problem can be addressed (to a certain extent) by exploiting the data’s longitudinal nature and the persistence of conditions like obesity in affecting care use. Suppose that current obesity is a function of lagged obesity, plus the change in obesity between the lagged and current periods, e.g., $W_{it}=\Delta W_{it}+W_{it-1}.$ Substituting for $W_{it}$ in (2) (and also for $X_{it}$ in the same way), we then estimate the model:

|  | $y_{itj}=\beta_{0}+\beta_{1}W_{it-1}+\beta_{2}X_{it-1}\left( W_{it-1} \right)+\epsilon_{it}\left( Z_{it}\left( W_{it}\left( W_{it-1} \right) \right),\Delta W_{it},\Delta X_{it}, \right)$ | (3) |
| --- | --- | --- |

The endogeneity problem likely would be reduced, depending on the extent of the correlation between current unobserved factors $Z_{it}$ and lagged obesity. Where a subset of current unobserved variables, $Z_{it}^{W},$ causally affect current obesity, $W_{it}$ and the need for long term care, this potential endogeneity problem is mitigated if lagged obesity/physical activity variables ($W_{it-1})$are used. For example, if the person’s current level of self-confidence is unobserved, and this leads to a need for care and also affects current obesity, then previous obesity rates are not endogenous. The problem remains if unobserved variables (e.g., self-confidence, stress, etc.) exert a historical effect on lagged obesity, in which this lagged effect also perpetuates direct impacts on current care use. Where time-invariant factors are unobserved, this could cause bias. In theory, a fixed-effects approach also would reduce this endogeneity issue. The problem is that obesity rates also are largely time-invariant, providing relatively few cases (in which a change in obesity status has occurred) with which to work. Furthermore, with multinomial models, many observations will be predicted perfectly, again substantially limiting number of valid cases.

The feasible set of outcomes $y_{itj}$ in the general (older) population includes the use of various types of long-term care support, no support, non-response and death. We estimate the model using multinomial logistic regression, which allows us to account for this range of outcomes simultaneously:

|  | $ln\left( \frac{p_{itj}}{p_{it1}} \right)=\beta_{0j}+{W_{it-1}\beta}_{1j}+X_{it-1}\beta_{xj}+\varepsilon_{it}$ | (4) |
| --- | --- | --- |

in which $j$ refers to the category corresponding to the mode of care, and $p_{itj}=prob\left( y_{itj}\left| X_{it-1},W_{it-1} \right. \right)$is the probability that the individual experiences outcome $j$. By focusing on future care use, we are relating current-wave obesity status to care use in in the next wave of data.

# Appendix C: Additional Statistical Tables

Table C1: Summary Statistics for control variables (measured at t-1)

|  | Whole sample | No care | Informal care (only) | Informal and privately paid care | Formal (care home/ LA care) | Non-response | Died |
| --- | --- | --- | --- | --- | --- | --- | --- |
|  | (1) | (2) | (3) | (4) | (5) | (6) | (7) |
| No. of obs. | 12323 | 7041 | 2504 | 347 | 187 | 1561 | 683 |
| Physical Exercise | 0.84 | 0.93*** | 0.72*** | 0.71*** | 0.46*** | 0.79*** | 0.55*** |
| Alcohol Drinking | 0.86 | 0.89*** | 0.81*** | 0.84 | 0.64*** | 0.82*** | 0.77*** |
| Smoked Ever | 0.63 | 0.62*** | 0.65* | 0.57** | 0.63 | 0.65* | 0.72*** |
| Smokes Now | 0.11 | 0.10*** | 0.11 | 0.07** | 0.13 | 0.13** | 0.16*** |
| Female | 0.55 | 0.51** | 0.64** | 0.80** | 0.73** | 0.56 | 0.44** |
| No Educ Qualif | 0.46 | 0.39** | 0.54** | 0.40* | 0.59** | 0.58** | 0.59** |
| Non-white | 0.01 | 0.01 | 0.01 | 0.01 | 0.01 | 0.03** | 0.01 |
| Age | 73.87 | 72.37** | 75.17** | 78.57** | 81.13** | 74.20* | 79.40** |
|  | [6.91] | [5.87] | [6.99] | [6.92] | [9.22] | [7.21] | [8.74] |
| Married | 0.57 | 0.59** | 0.57 | 0.27** | 0.17** | 0.61** | 0.45** |
| Number of | 2.22 | 2.20* | 2.42** | 1.83** | 1.89** | 2.21 | 2.08* |
| Children | [1.53] | [1.45] | [1.61] | [1.48] | [1.77] | [1.59] | [1.69] |
| Living Alone | 0.29 | 0.26** | 0.28 | 0.55** | 0.60** | 0.27 | 0.40** |
| Working | 0.03 | 0.04** | 0.01** | 0.01* | 0.00* | 0.03 | 0.00** |
| Homeowner | 0.73 | 0.78** | 0.68** | 0.75 | 0.51** | 0.66** | 0.61** |
| Real Per Capita Total HH | 149.45 | 177.05** | 107.35** | 167.42 | 83.09** | 114.78** | 107.47** |
| Wealth, 100K | [211.84] | [241.61] | [148.24] | [180.64] | [103.66] | [171.38] | [143.83] |
| Real Per Capita | 10.48 | 11.56** | 8.91** | 10.37 | 9.28* | 8.90** | 9.05** |
| HH Total Income, 1K | [8.49] | [9.53] | [6.22] | [6.60] | [4.67] | [6.75] | [7.80] |
| ADL count | 0.46 | 0.18** | 0.94** | 0.89** | 1.51** | 0.56** | 0.95** |
|  | [0.99] | [0.54] | [1.29] | [1.30] | [1.52] | [1.13] | [1.38] |
| IADL count | 0.43 | 0.14** | 0.89** | 0.96** | 1.45** | 0.51** | 1.07** |
|  | [0.88] | [0.44] | [1.14] | [0.99] | [1.20] | [0.96] | [1.30] |
| Mobility | 2.32 | 1.39** | 4.00** | 4.35** | 5.26** | 2.45* | 3.75** |
| Limitations Count | [2.48] | [1.79] | [2.58] | [2.56] | [2.58] | [2.60] | [2.71] |
| High Blood Pressure | 0.47 | 0.44** | 0.54** | 0.60** | 0.63** | 0.48 | 0.49 |
| Diabetes | 0.10 | 0.08** | 0.13** | 0.17** | 0.14* | 0.11 | 0.14** |
| Cancer | 0.09 | 0.09** | 0.09 | 0.11 | 0.12 | 0.08* | 0.20** |
| Lung Disease | 0.08 | 0.05** | 0.11** | 0.10 | 0.17** | 0.08 | 0.16** |
| Heart Disease | 0.26 | 0.20** | 0.34** | 0.36** | 0.40** | 0.27 | 0.40** |
| Stroke | 0.06 | 0.04** | 0.10** | 0.11** | 0.17** | 0.07 | 0.12** |
| Psychiatric Problems | 0.06 | 0.05** | 0.07** | 0.11** | 0.06 | 0.05 | 0.05 |
| Arthritis | 0.42 | 0.34** | 0.60** | 0.64** | 0.58** | 0.41 | 0.43 |

Notes: ** indicates that the average for a specific category is statistically different from the average for the whole sample at a 1% level of significance, * - at the 5% level and + - at the 10% level.

Table C2: Relative Risk Ratios from Multinomial Logit – No controls, Partial Controls A

|  | No controls | | | Partial controls A | | |
| --- | --- | --- | --- | --- | --- | --- |
|  | Any care | Non-response | Died | Any care | Non-response | Died |
|  | (1) | (2) | (3) | (4) | (5) | (6) |
| Underweight | 1.78* | 1.37 | 3.05** | 1.57+ | 1.21 | 2.45** |
|  | (0.42) | (0.41) | (0.87) | (0.37) | (0.37) | (0.73) |
| Overweight | 0.93 | 0.82** | 0.68** | 0.96 | 0.85* | 0.73** |
|  | (0.06) | (0.06) | (0.06) | (0.06) | (0.06) | (0.07) |
| Obese | **1.75**** | 1.13 | 0.80* | **1.65**** | 1.1 | 0.75* |
|  | (0.12) | (0.09) | (0.09) | (0.11) | (0.09) | (0.09) |
| Physical Activity |  |  |  | 0.20** | 0.31** | 0.10** |
|  |  |  |  | (0.01) | (0.02) | (0.01) |
| Drink |  |  |  | 0.53** | 0.57** | 0.48** |
|  |  |  |  | (0.04) | (0.05) | (0.05) |
| Smoked ever |  |  |  | 1.11+ | 1.15* | 1.59** |
|  |  |  |  | (0.06) | (0.07) | (0.15) |
| Smoke now |  |  |  | 0.87 | 1.02 | 1.01 |
|  |  |  |  | (0.08) | (0.1) | (0.13) |
|  |  |  |  |  |  |  |
| Wave=2 | 0.57** | 0.47** | 0.40** | 0.60** | 0.49** | 0.43** |
|  | (0.03) | (0.03) | (0.04) | (0.03) | (0.03) | (0.05) |
| Wave=4 | 0.53** | 0.25** | 0.42** | 0.55** | 0.26** | 0.45** |
|  | (0.03) | (0.02) | (0.04) | (0.03) | (0.02) | (0.05) |
| Observations | 12,323 | | | 12,323 | | |
| Pseudo R2 | 0.0267 | | | 0.0737 | | |

Notes: All regressions include time dummies, and standard errors are clustered at individual levels. ** indicates significance at 1% level, * at 5% level and + at 10% level.

Table C3: Relative Risk Ratios From Multinomial Logit – Partial Controls B, Full Controls

|  | Partial controls B | | | Full controls | | |
| --- | --- | --- | --- | --- | --- | --- |
|  | Any care | Non-response | Died | Any care | Non-response | Died |
|  | (1) | (2) | (3) | (4) | (5) | (6) |
| Underweight | 1.32 | 1.2 | 2.50** | 1.36 | 1.18 | 2.33* |
|  | (0.35) | (0.38) | (0.82) | (0.36) | (0.38) | (0.78) |
| Overweight | 0.98 | 0.84* | 0.77* | 0.96 | 0.83* | 0.77* |
|  | (0.06) | (0.06) | (0.08) | (0.06) | (0.06) | (0.08) |
| Obese | **1.28**** | 0.94 | 0.79+ | **1.25**** | 0.93 | 0.79+ |
|  | (0.09) | (0.08) | (0.1) | (0.09) | (0.08) | (0.1) |
| Physical Activity | 0.62** | 0.57** | 0.34** | 0.62** | 0.58** | 0.35** |
|  | (0.05) | (0.05) | (0.04) | (0.05) | (0.05) | (0.04) |
| Drink | 0.84* | 0.75** | 0.67** | 0.86* | 0.76** | 0.70** |
|  | (0.06) | (0.06) | (0.08) | (0.07) | (0.07) | (0.08) |
| Smoked ever | 1.06 | 1.1 | 1.26* | 1.04 | 1.09 | 1.18 |
|  | (0.06) | (0.08) | (0.13) | (0.06) | (0.07) | (0.12) |
| Smoke now | 0.91 | 0.98 | 1.40* | 0.93 | 0.99 | 1.42* |
|  | (0.08) | (0.1) | (0.2) | (0.09) | (0.1) | (0.2) |
| Female | 1.42** | 1.15* | 0.51** | 1.44** | 1.18* | 0.55** |
|  | (0.09) | (0.08) | (0.05) | (0.09) | (0.08) | (0.06) |
| No Educ Qualif | 0.96 | 1.44** | 1.26* | 0.98 | 1.45** | 1.30** |
|  | (0.06) | (0.1) | (0.12) | (0.06) | (0.1) | (0.13) |
| Non-white | 0.79 | 1.68* | 0.87 | 0.81 | 1.69* | 0.99 |
|  | (0.19) | (0.38) | (0.37) | (0.19) | (0.38) | (0.42) |
| Age | 1.06** | 1.04** | 1.11** | 1.06** | 1.04** | 1.11** |
|  | 0 | (0.01) | (0.01) | 0 | (0.01) | (0.01) |
| Married | 1.19* | 1.43** | 0.99 | 1.20* | 1.43** | 1.01 |
|  | (0.1) | (0.14) | (0.13) | (0.1) | (0.14) | (0.14) |
| Number of Children | 1.04* | 0.98 | 0.97 | 1.03+ | 0.98 | 0.96 |
|  | (0.02) | (0.02) | (0.03) | (0.02) | (0.02) | (0.03) |
| Living Alone | 0.76** | 0.78* | 0.81 | 0.75** | 0.77* | 0.81 |
|  | (0.07) | (0.08) | (0.11) | (0.07) | (0.08) | (0.11) |
| Working | 0.67+ | 1.29 | 0.45 | 0.72 | 1.32 | 0.51 |
|  | (0.14) | (0.23) | (0.27) | (0.15) | (0.24) | (0.31) |
| Homeowner | 1.05 | 0.76** | 0.84+ | 1.07 | 0.76** | 0.82+ |
|  | (0.07) | (0.06) | (0.09) | (0.07) | (0.06) | (0.09) |
| Real per Capita Total HH Wealth | 1.00** | 1.00* | 1.00 | 1.00 | 1.00+ | 1.00 |
| 100K | 0.00 | 0.00 | 0.00 | 0.00 | 0.00 | 0.00 |
| Real per Capita HH Total Income | 1.00* | 1.00+ | 1.00+ | 1 | 1.00* | 1.00+ |
| 1K | 0.00 | 0.00 | 0.00 | 0.00 | 0.00 | 0.00 |
| ADL Count | 1.15** | 1.26** | 1.14* | 1.15** | 1.26** | 1.14* |
|  | (0.05) | (0.06) | (0.06) | (0.05) | (0.06) | (0.06) |
| IADL Count | 1.79** | 1.62** | 1.95** | 1.76** | 1.60** | 1.89** |
|  | (0.09) | (0.08) | (0.12) | (0.09) | (0.08) | (0.11) |
| Mobility limitations | 1.38** | 1.08** | 1.24** | 1.32** | 1.06** | 1.20** |
| Count | -0.02 | -0.02 | -0.03 | -0.02 | -0.02 | -0.03 |
| High blood pressure |  |  |  | 1.12* | 1.06 | 1.07 |
|  |  |  |  | (0.06) | (0.07) | (0.1) |
| Diabetes |  |  |  | 1.15 | 1.05 | 1.30+ |
|  |  |  |  | (0.10) | (0.11) | (0.18) |
| Cancer |  |  |  | 1.04 | 1.01 | 2.82** |
|  |  |  |  | (0.09) | (0.11) | (0.35) |
| Lung disease |  |  |  | 1.31* | 1.15 | 1.75** |
|  |  |  |  | (0.14) | (0.14) | (0.26) |
| Heart disease |  |  |  | 1.30** | 1.24** | 1.64** |
|  |  |  |  | (0.08) | (0.09) | (0.16) |
| Stroke |  |  |  | 1.44** | 1.28+ | 1.35+ |
|  |  |  |  | (0.17) | (0.17) | (0.22) |
| Psychiatric disorders |  |  |  | 1.23+ | 1.03 | 0.97 |
|  |  |  |  | (0.15) | (0.15) | (0.21) |
| Arthritis |  |  |  | 1.41** | 1.07 | 0.93 |
|  |  |  |  | (0.08) | (0.07) | (0.09) |
| Wave=2 | 1.83** | 3.38** | 2.23** | 1.98** | 3.46** | 2.50** |
|  | (0.12) | (0.29) | (0.26) | (0.13) | (0.3) | (0.3) |
| Wave=4 | 0.93 | 1.65** | 0.85 | 0.95 | 1.66** | 0.9 |
|  | (0.06) | (0.15) | (0.11) | (0.07) | (0.15) | (0.12) |
| Observations | 12,323 | | | 12,323 | | |
| Pseudo R2 | 0.1786 | | | 0.1841 | | |

Notes: All regressions include time dummies, and standard errors are clustered at the individual level. ** indicates significance at 1% level, * at 5% level and + at 10% level.

# Appendix D: Indicative Estimates of the Obesity Epidemic’s Costs

We start with a base year of 2009 – the last year of our sample with data on obesity – and consider the impact two years later (2011) – in accordance with our analytic methodology. The proportion of people who are obese in 2009 and use care in 2011 is around 1.25 times greater than the proportion of non-obese people in 2009 who use care in 2011, according to our estimates. The excess number due to previous obesity can be determined as the difference between the number of people who previously were obese (but were not care users), assuming a 1.25 greater obesity effect and the number as though no such effect exists. The relative risk ratio $(rrr)$ is:

|  | $rrr=\frac{\pi^{1}}{\pi^{0}}=\frac{\pi^{0}\beta}{\pi^{0}}=\beta=1.25$ | (5) |
| --- | --- | --- |

in which $\pi^{k}=\frac{N_{1k}^{t}}{N_{1k}^{t}+N_{0k}^{t}}$, and $N_{jk}^{t}$ is the population (at time $t$) who either use care or do not use any, denoted as $j=0,1$ and obesity or not, denoted as $k=0,1$. Accordingly, we can project the number of people at time $t$ who will be using care relying on the estimate of $\pi^{k}$ and assuming that this rate remains unchanged over time:

|  | $N_{1k}^{t}=N_{0k}^{t}\left( \frac{\pi^{k}}{1-\pi^{k}} \right)=N_{0k}^{t-1}\left( \frac{\pi^{k}}{1-\pi^{k}} \right)$ | (6) |
| --- | --- | --- |

Moreover, the excess effect from obesity is the difference, $\Delta^{t},$ between the projected number of people with care needs and (previous) obesity when (a) applying the estimated obesity effect rate $\pi^{1}$ and (b) assuming no obesity effect, i.e., applying $\pi^{0}$:

|  | $\Delta^{t}=N_{11}^{t}-N_{11}^{t\pi^{0}}=N_{01}^{t-1}\left( \frac{\pi^{1}}{1-\pi^{1}} \right)-N_{01}^{t-1}\left( \frac{\pi^{0}}{1-\pi^{0}} \right)$  $=N_{01}^{t-1}\pi^{0}\left( \frac{\beta}{1-\beta\pi^{0}}-\frac{1}{1-\pi^{0}} \right)=N_{01}^{t-1}\pi^{0}\frac{\beta-1}{\left( 1-\beta\pi^{0} \right)\left( 1-\pi^{0} \right)}$ | (7) |
| --- | --- | --- |

This calculation assumes no differential mortality rate between obese and non-obese people (an assumption that is largely consistent with our results above). The no-obesity-effect rate, $\pi^{0},$ is assumed to be the observed proportion of people in the ELSA sample in 2009 who used care, *but were not obese*, a rate of $\pi^{0}=0.175$. We use our estimation results of $\beta=1.25$. The previous number of people who were obese and did not use care also is based on sample estimates from our data. In particular, we observe that around 20.78% of older people in the 2009 sample wave were obese, but not using care.^[[1]](#footnote-1)^ We apply this rate to England’s population, giving $N_{01}^{t-1}$ = 1.799 million people in this category, to calculate the excess effect in 2011.

This calculation also can be repeated for later pairs of years, e.g., 2013 compared with 2011. In this case, we uprate our starting value for the number of older people in 2011 who were obese, but not receiving care, using the projected changes in obesity rates as produced by Foresight (2007) (an average of 1.67% over a two-year period). We assume that this figure applies equally to all ages. Population change is based on the ONS population projections.^[[2]](#footnote-2)^

Table D1 shows this projection’s results. We start with 20.78% of the age 65-and-up population who are obese and not using any care in row 4 for 2009 (a 1.67% biennial increase is applied to this number to obtain the corresponding share for 2013). Combining information from row 3 and row 4, we obtain the size of the obese population among those ages 65 and up who do not use any care in the current year (row 5).

Row 6 applies equation (7) to calculate $\Delta^{t}$, i.e., the number of people who, over the two-year period, developed a need for informal care because they were obese, holding all other things constant. This ‘excess’ number of people in need of informal care corresponds to about 7.29% of the total number of informal care users.

We can estimate a cost associated with this excess effect by calculating a unit cost of informal care, as follows. First, we take estimates from our sample on the share of informal care users in the 65-and-up population, 21.90% in year 2009, and apply this share to the size of the older population and arrive at estimates of the numbers of informal care users in row 7. Second, we deflate the aforementioned estimate of the value of informal care: £55 billion in 2011 to £53.3 billion in 2009. Together, these figures provide the average value of informal care per care user in the amount of £28,410 per year (row 9).

Key financial results from the projections are provided in rows 10 and 12 in Table 5 (with corresponding percentages in rows 11 and 12). Applying the 2009 unit value of informal care to the numbers in row 6 (assuming no inflation and no wage growth) provides the estimate of the annual value of informal care linked to past obesity (row 10). In year 2011, it is calculated to be £3.9 billion, with a value of £4.3 billion in 2013. This amount can be interpreted as the excess use of informal care, which could have been avoided if obesity were addressed in people who did not use care two years ago. In other words, if the cost of addressing obesity via public health interventions among the 65-and-up population group was up to £3.9 billion in 2011, this would still represent an overall cost savings from a societal perspective. For a comparison, Scarborough et al. [36] estimate the direct cost of both overweight and obesity to the NHS at £5.1 billion per year. Another comparison is made with the Public Health England budget: Programmes tackling obesity are funded from a ring-fenced, local authority grant, which in year 2015-2016 totalled £3.4 billion [37] and was not limited to obesity-focussed interventions or to the elderly population.

The second notable result is the additional cost of increasing obesity rates over time. All other things being equal, in 2009, obesity rates are associated with the excess number of informal care users is 137,000 in 2011. But starting in 2011, the equivalent figure is higher, at 151,000. This increase can be expressed in monetary terms (row 12), with the following interpretation: If obesity rates were halted at the 2009 level (for 2011), the cost of informal care would have been almost £400 million less two years later in 2011. In other words, halting further increases in obesity would have saved a projected £200 million per year in care costs.

These projections in cost terms are particularly sensitive to the assumed unit value of informal care. For example, using a value of half that in the tables would reduce all other financial figures by half. However, the results indicate the effect’s considerable magnitude, given reasonable assumptions.

Table D1: Estimates of obesity epidemics’ future costs in terms of informal care’s value

|  |  | 2009 | 2011 | 2013 |
| --- | --- | --- | --- | --- |
| 1 | Total population, 000 | 52,640 | 53,110 | 53,870 |
| 2 | % 65 plus | 16.27% | 16.44% | 17.27% |
| 3 | Population 65 plus, 000 | 8,565 | 8,731 | 9,303 |
| 4 | % obese among 65 plus, no care use | 20.78% | 22.45% | 24.12% |
| 5 | Obese population 65 plus, no care use ($N_{01}^{t-1}$) | 1,779,917 | 1,960,386 | 2,244,194 |
| 6 | Excess number of informal care users due to obesity, compared with previous period |  | 136,671 | 150,528 |
| 7 | Informal care users 65 plus (2009) | 1,875,970 | 7.29% | 8.02% |
| 8 | Value of informal care per year, 000 GBP | 53,300,000 |  |  |
| 9 | Average annual value of informal care per care user, GBP | 28410 |  |  |
| 10 | Value of informal care per year linked to past obesity, 000 GBP |  | £3,882,813 | £4,276,497 |
| 11 | Value of informal care linked to past obesity, % of total |  | 7.28% | 8.02% |
| 12 | Two-year (annual) increase in value of informal care linked to obesity epidemic, 000 GBP |  |  | £393,684  (£196,842) |
| 13 | Two-year (annual) increase in value of informal care linked to obesity epidemic, % total |  |  | 0.74%  (0.37%) |

1. The overall obesity rate, based on our sample, is 29.59%, slightly higher than the estimate of 27.88%, based on the 2009 Health Survey of England. [↑](#footnote-ref-1)
2. Office for National Statistics: National Population Projections: 2014-based Extra Variants Report. Accessed online at https://www.ons.gov.uk/peoplepopulationandcommunity/populationandmigration/populationprojections/articles/nationalpopulationprojections/2014basedextravariantsreport#appendix-c-charts-population-aged-65-and-over-for-the-constituent-countries on on June 21, 2019 (2015). [↑](#footnote-ref-2)
